# Supplementary material for: Identification of photoperiod-induced specific miRNAs in the adrenal glands of Sunite sheep (Ovis aries)
Source: Front Vet Sci. 2022 Jul 22;9:888207. doi: 10.3389/fvets.2022.888207 (PMC9354845; doi:10.3389/fvets.2022.888207)
Supplement: Supplementary file 1 [file Data_Sheet_1.ZIP › Supplementary file/miRDeep2 file.zip!/miRDeep2 file/Novel_Structure/SPLP42A1/Novel_429.pdf]

Provisional ID	:	NC_019459.2_4810
Score total	:	75
Score for star read(s)	:	3.9
Score for read counts	:	67.4
Score for mfe	:	2.1
Score for randfold	:	1.6
Score for cons. seed	:	
Total read count	:	144
Mature read count	:	132
Loop read count	:	0
Star read count	:	12

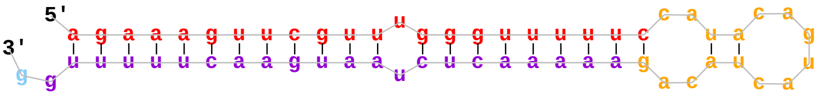

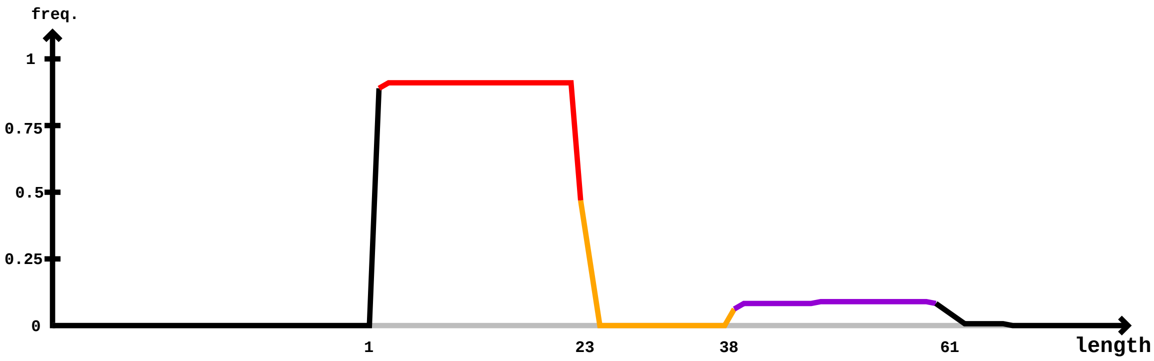

Mature Star

```
5'- gacuuucuagaccauaugauuuuggguuggccagaaaguucguuuggguuuuuccauacaguacuacagaaaaacucuaaugaacuuuuuggccaacccuauaguaaaug  -3'   obs
    gacuuucuagaccauaugauuuuggguuggccagaaaguucguuuggguuuuuccauacaguacuacagaaaaacucuaaugaacuuuuuggccaacccuauaguaaaug        exp
    .......................(((((((((((((((((((((.(((((((((..((......))..))))))))).)))))))))))))))))))))...........     reads     mm       sample
    ................................agaaaguucguuuggguuuuu.........................................................     1         0        seq
    ................................aAaaaguucguuuggguuuuu.........................................................     62        1        seq
    ................................aAaaaguucguuuggguuuuuc........................................................     66        1        seq
    .................................gaaaguuUguuuggguuuuu.........................................................     1         1        seq
    .................................gaaaguuUguuuggguuuuuc........................................................     1         1        seq
    .................................gaaaguucguuuggguuuuuc........................................................     1         0        seq
    .....................................................................aaaaacucAaaugaacuuuuu....................     1         1        seq
    .....................................................................aaaaacucAaaugaacuuuuug...................     8         1        seq
    ......................................................................aaaacucAaaugaacuuuuug...................     3         1        seq
    .............................................................................aaugaacuuuuuggccaacc.............     1         0        seq
```
